# Supplementary figures and images for: Identifying enhancer properties associated with genetic risk for complex traits using regulome-wide association studies
Source: PLoS Comput Biol. 2022 Sep 7;18(9):e1010430. doi: 10.1371/journal.pcbi.1010430 (PMC9484640; doi:10.1371/journal.pcbi.1010430)

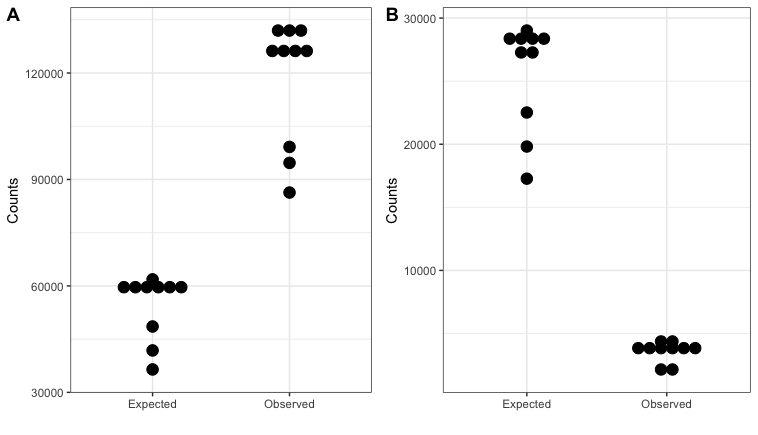

Supplement: S1 Fig — Observed vs expected epigenetic mark ChIP-seq peak overlaps of 10 chromHMM brain enhancers A) Enhancer marker H3K27ac peaks from middle frontal area 46 are enriched in chromHMM brain enhancers B) Heterochromatin marker H3K9me3 peaks from middle frontal area 46 are depleted in chromHMM brain enhancers. (TIFF) [file pcbi.1010430.s001.tiff]

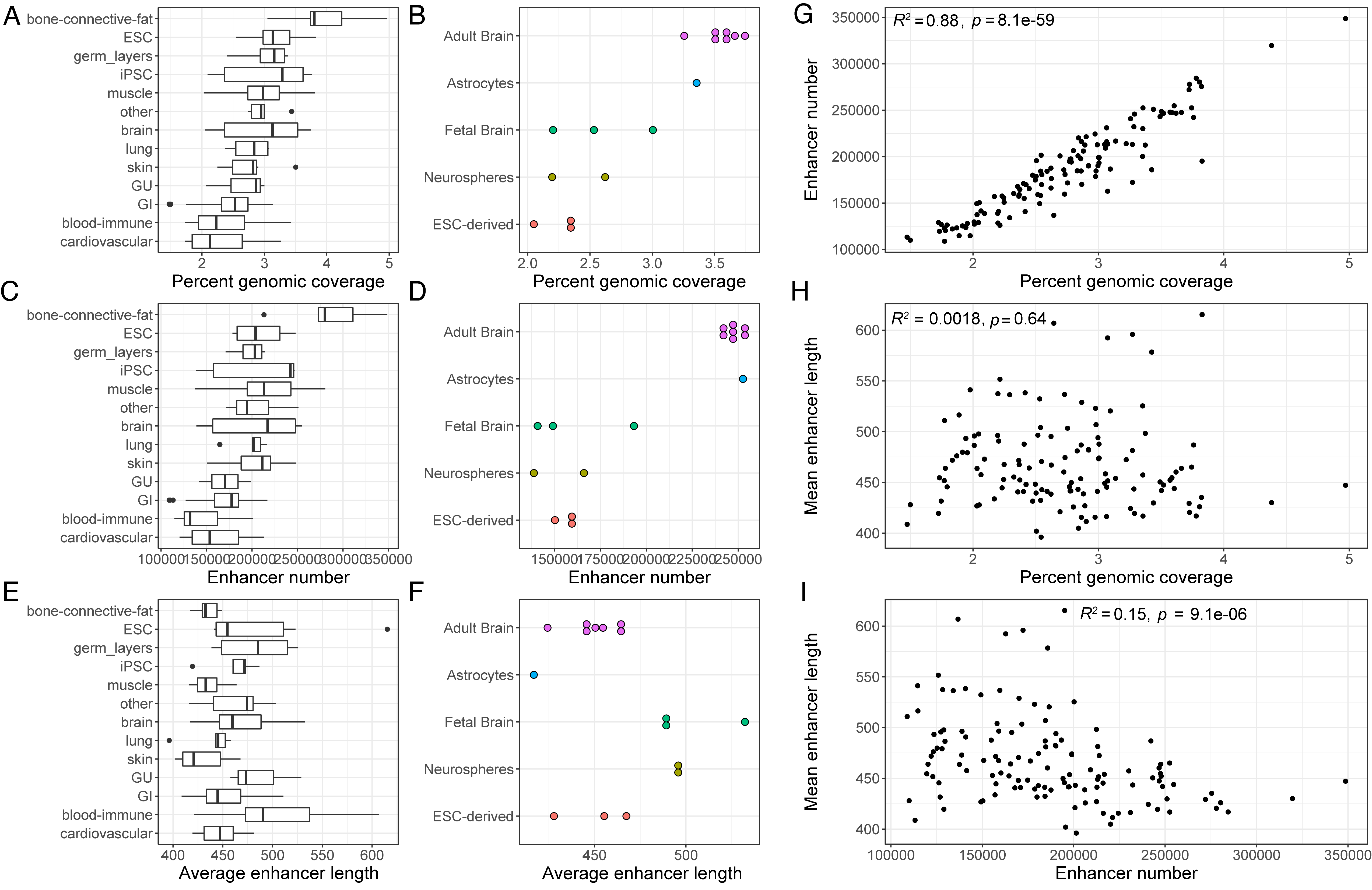

Supplement: S2 Fig — A) Genomic coverage by tissue category. B) Adult brain and astrocyte enhancer annotations had the highest genomic coverage compared to fetal, neurosphere, and ESC-derived enhancer annotations. C) Enhancer number by tissue category. D) Adult brain and astrocyte enhancer annotations had the highest enhancer number compared to fetal, neurosphere, and ESC-derived enhancer annotations. E) Enhancer length by tissue category. F) Fetal brain and neurosphere enhancer annotations had the highest mean enhancer length compared to adult brain, astrocyte, and ESC-derived enhancer annotations. G) Enhancer number and percent genomic coverage are tightly associated (p = 8.1E-59). H) Enhancer length and genomic coverage are not associated (p = 0.64). I) Enhancer length and enhancer number are negatively correlated (p = 9.1E-6). (TIF) [file pcbi.1010430.s002.tif]

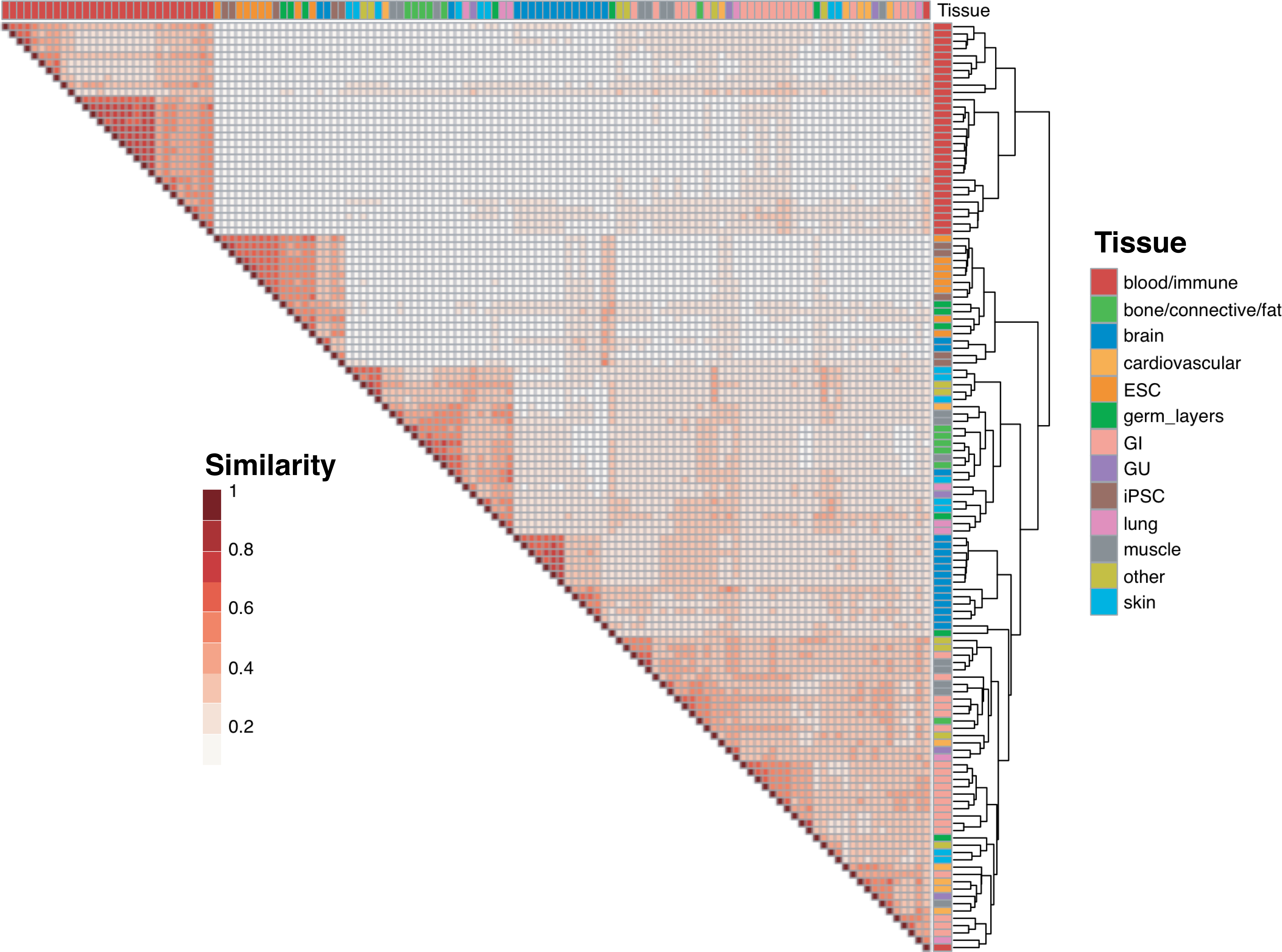

Supplement: S3 Fig — Annotations are arranged by hierarchical clustering. Differential enhancer utilization between tissues clusters samples by organ. Brain enhancers largely cluster together, with the exception of ESC-derived cells and astrocytes. Interestingly, while the female fetal brain and male fetal brain were in the same cluster, the female fetal brain enhancers were slightly more similar to the neurosphere samples than to the male fetal brain samples. This is likely due to technical differences, as fetal male brain (E081) is the only sample from this cluster where the primary tissue was from the Broad Institute, while all other neurosphere/fetal samples were from UCSF. The differences in sample origin did not have a major effect on the overall cluster structure, as E081 did not cluster with any of the other Broad Institute samples such as H9 derived neuronal progenitor cultured cells (E009), H9 derived neuron cultured cells (E010), or NH-A Astrocytes (E125). (TIF) [file pcbi.1010430.s003.tif]

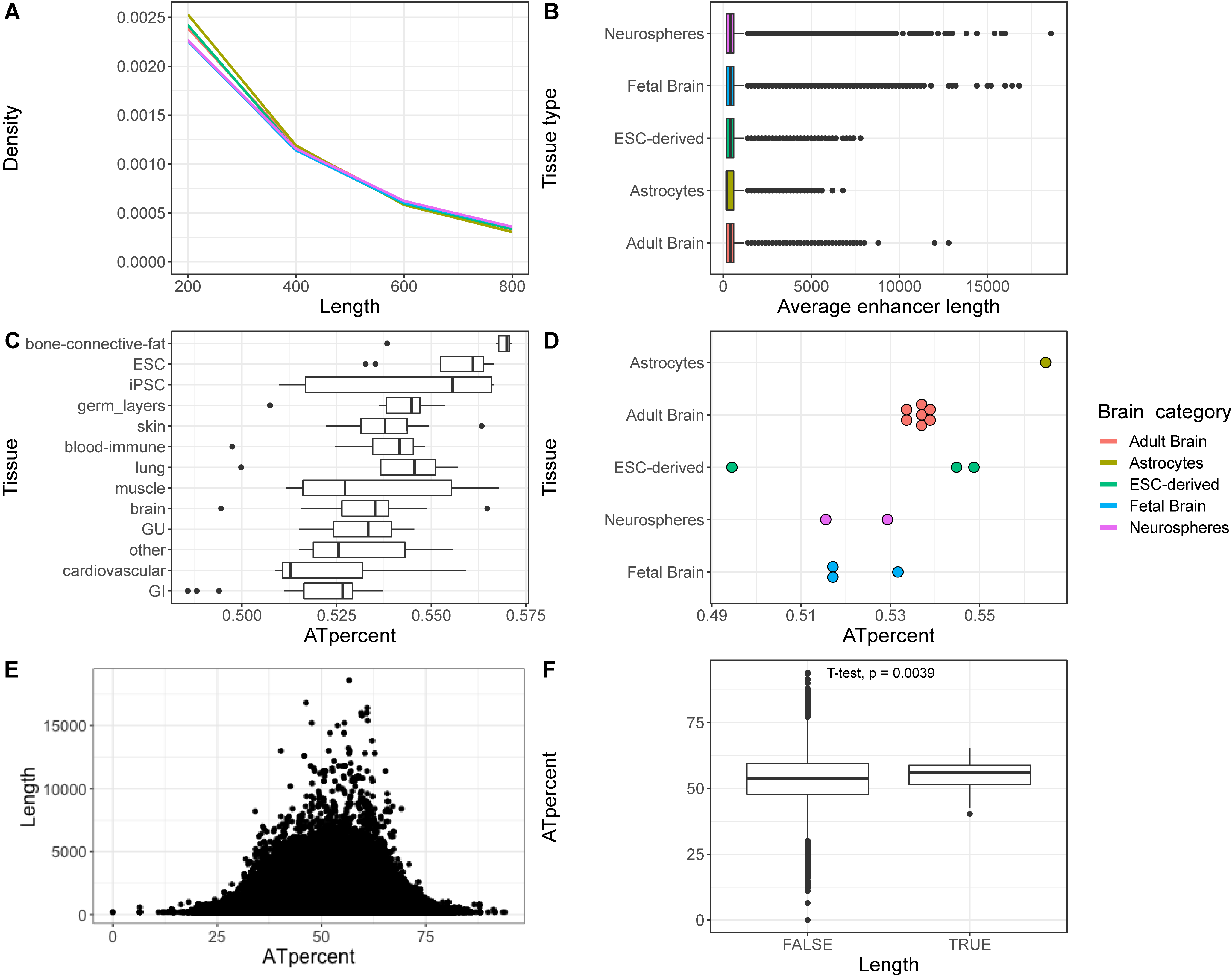

Supplement: S4 Fig — A) Fetal brain and neurosphere enhancer annotations are underrepresented in the lowest length bins compared to other brain samples. B) Fetal brain and neurosphere enhancer annotations have more super-long enhancers than other brain enhancer annotations. C) AT percentage of enhancer annotations by tissue. D) Adult brain enhancer annotations have slightly higher AT richness compared to fetal brain and neurosphere enhancer annotations. E-F) On average, super-enhancers in the brain (>10 kb, ‘TRUE”) tend to be more AT rich than enhancers of shorter length (“FALSE”). (TIF) [file pcbi.1010430.s004.tif]

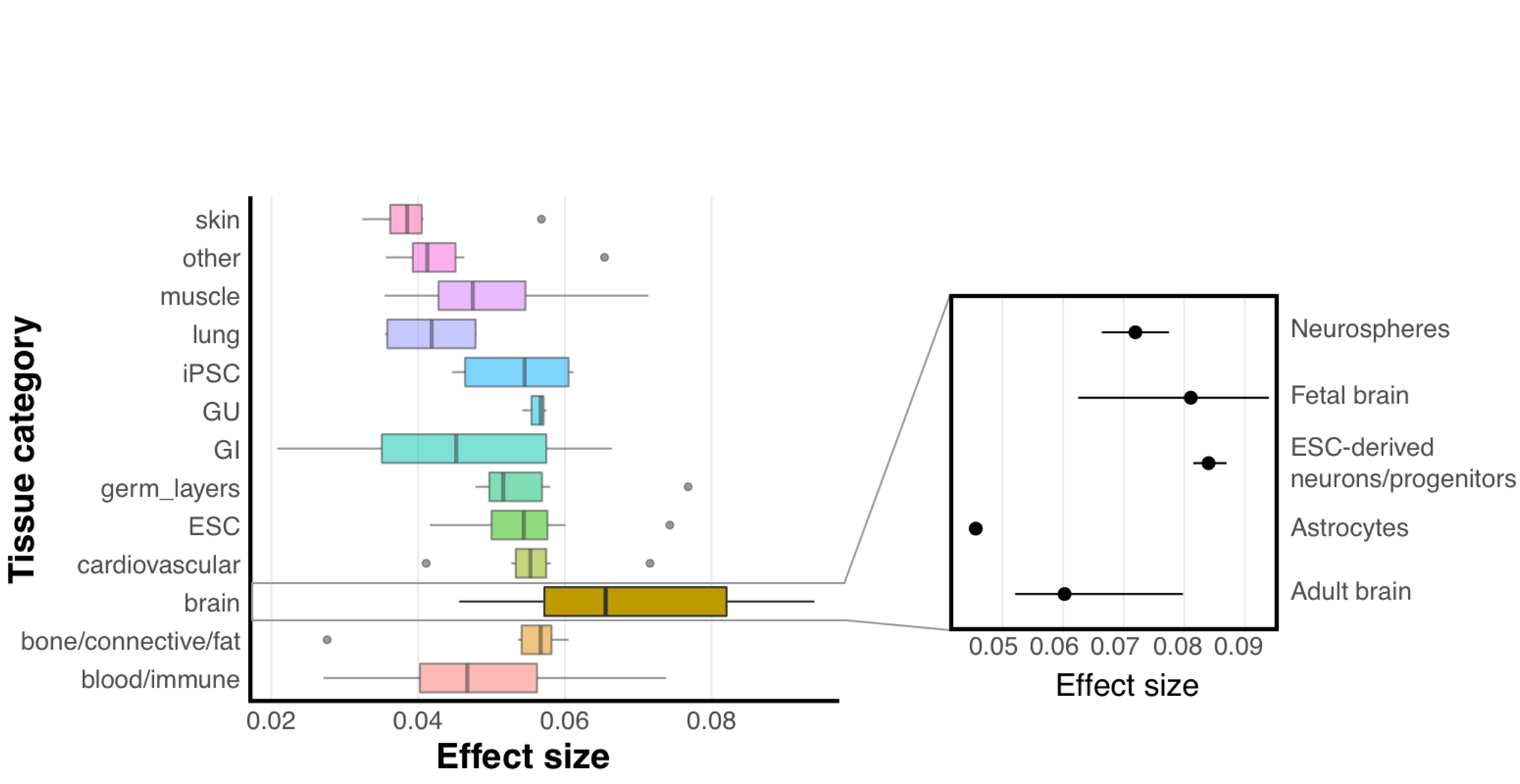

Supplement: S5 Fig — Brain enhancers had the strongest association, with germinal matrix (E070) having the most associated individual annotation. (TIF) [file pcbi.1010430.s005.tif]

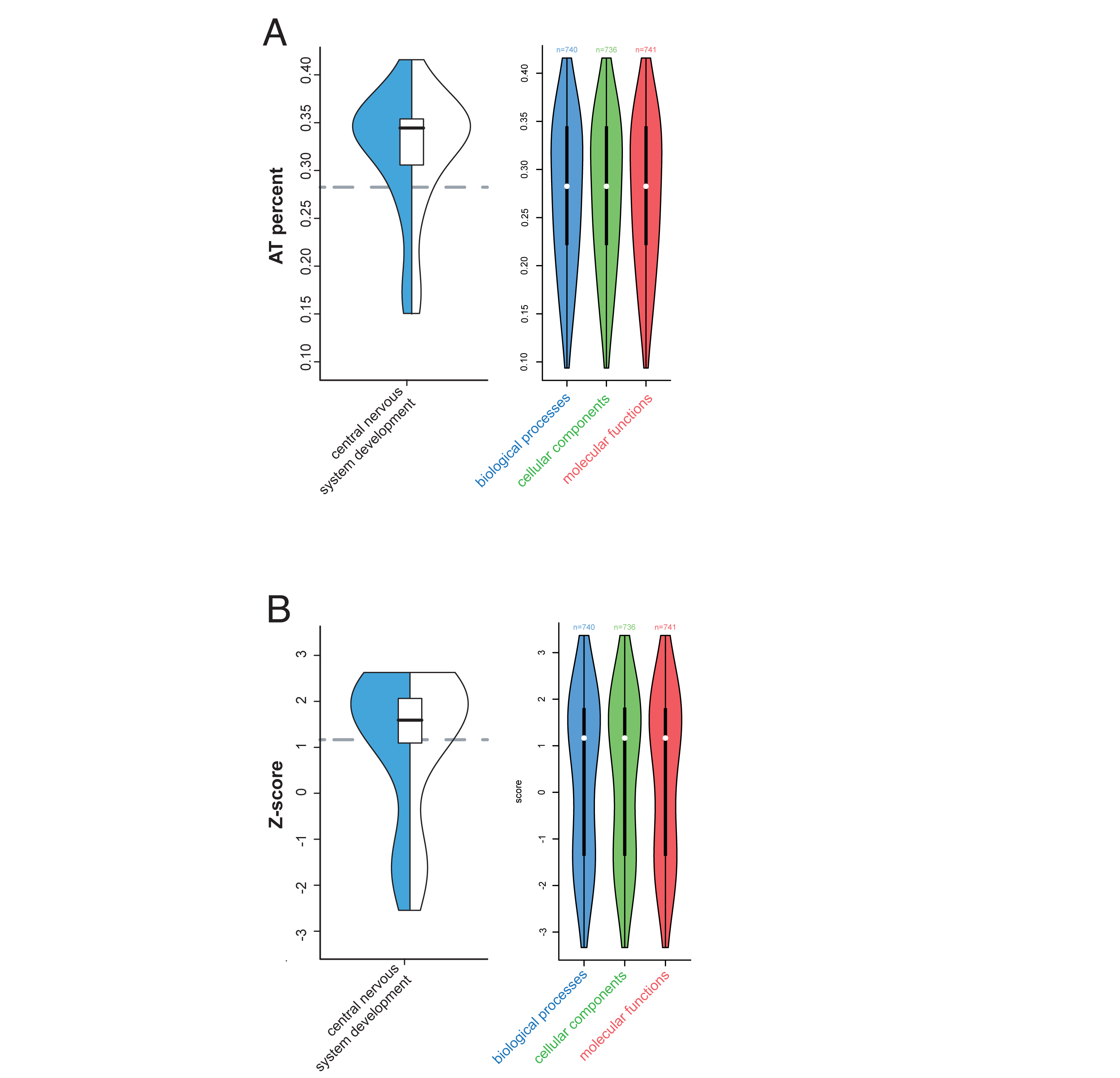

Supplement: S6 Fig — A) Higher median motif AT percentage of a given TF is positively associated with the TF being annotated to the Gene Ontology term “cell morphogenesis during neuron differentiation” but not general GO terms (biological processes, cellular components, molecular function) B) TFs with higher median Z-score in the RWAS analysis are more likely to be annotated to “cell morphogenesis during neuron differentiation” and are not more likely to be annotated to general GO terms. (TIF) [file pcbi.1010430.s006.tif]

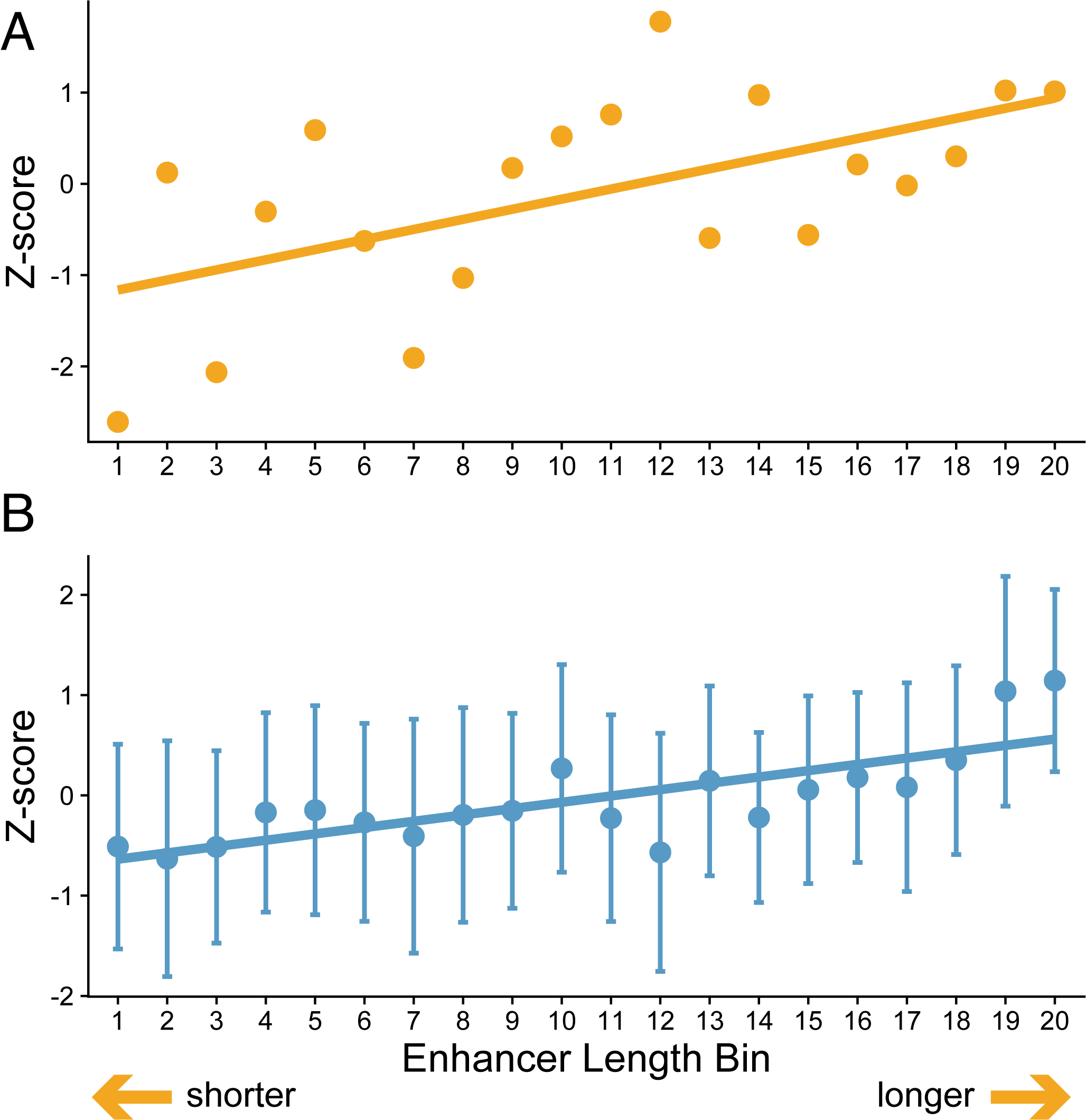

Supplement: S7 Fig — A. Z-scores are higher in longer enhancers compared to shorter enhancers in the PGC2 schizophrenia GWAS. B. Z-scores show similar inflation in long enhancers in 75 unrelated UK Biobank traits. (TIF) [file pcbi.1010430.s007.tif]
