# Supplementary material for: Identifying enhancer properties associated with genetic risk for complex traits using regulome-wide association studies
Source: PLoS Comput Biol. 2022 Sep 7;18(9):e1010430. doi: 10.1371/journal.pcbi.1010430 (PMC9484640; doi:10.1371/journal.pcbi.1010430)
Supplement: Attachment — Submitted filename: reviewer_comments_response_v3.docx [file pcbi.1010430.s012.docx]

**Responses to reviewers**

Thank you for the opportunity to respond to the critiques of our manuscript “Identification of enhancer properties associated with genetic risk for complex traits using Regulome-wide association.” We thank the reviewers for their valuable feedback and have addressed each of their concerns, as described below.

Reviewer 1

R1) 1. The authors used a highly comprehensive approach to define enhancers, particularly for the brain. But almost all data used were from ChIP-seq. I understand that those are good indicators of regulators overall, but the precision at individual genes might still be questionable. Given gene expression is the ultimate product of the regulation, why gene expression information was not considered. I would love to see one additional analysis to show how well the expression activity is related to the states of the predicted regulators. It might be a challenge to combine regulators across a long genomic region to predict expression. I'd like to know how the authors address this issue or their thoughts about this problem. In the end, I really hope to see that the enhancers are meaningful in relation to gene expression instead of using one histone pattern to prove another. I am not completely convinced about the current way defining enhancers.

Thanks for raising these points. An advantage of the ChromHMM models is that they have been extensively validated using many of the approaches that one might imagine to be informative. For instance, with respect to the specific question about relating enhancer locations to gene expression, Jason Ernst and Manolis Kellis published compelling data indicating that tissue-specific enhancers are strongly enriched near genes with tissue-specific expression (Ernst and Kellis, 2015. *Nat Biotechnology.* PMID: 25690853). We have added additional information and references explaining some of the previous validation of this resource: “Enhancers predicted by chromHMM have been well characterized with respect to their agreement with enhancers predicted by other methods, and tissue-specific ChromHMM enhancers are enriched near genes with matched tissue-specific expression” (line 156). Links between disease-associated enhancers and gene expression are more novel to our study. As detailed below, we have added a new analysis showing that schizophrenia-associated enhancers are enriched for eQTLs.

R1) 2. The authors used "enhancer" for the functional elements they defined in the paper. Are they really all enhancers? Please clarify. Would the other types of regulators be relevant too? or they just used enhancers to represent all regulators, including silencers and insulators.

The ChromHMM 25-state models used in our study are documented on the ROADMAP Epigenomics website at <https://egg2.wustl.edu/roadmap/web_portal/imputed.html#chr_imp>. The 25 states are as follows:

| **STATE NO.** | **MNEMONIC** | **DESCRIPTION** | **COLOR NAME** | **COLOR CODE** |
| --- | --- | --- | --- | --- |
| 1 | TssA | Active TSS | Red | 255,0,0 |
| 2 | PromU | Promoter Upstream TSS | Orange Red | 255,69,0 |
| 3 | PromD1 | Promoter Downstream TSS 1 | Orange Red | 255,69,0 |
| 4 | PromD2 | Promoter Downstream TSS 2 | Orange Red | 255,69,0 |
| 5 | Tx5 | Transcribed - 5' preferential | Green | 0,128,0 |
| 6 | Tx | Strong transcription | Green | 0,128,0 |
| 7 | Tx3 | Transcribed - 3' preferential | Green | 0,128,0 |
| 8 | TxWk | Weak transcription | Lighter Green | 0,150,0 |
| 9 | TxReg | Transcribed & regulatory (Prom/Enh) | Electric Lime | 194,225,5 |
| 10 | TxEnh5’ | Transcribed 5' preferential and Enh | Electric Lime | 194,225,5 |
| 11 | TxEnh3’ | Transcribed 3' preferential and Enh | Electric Lime | 194,225,5 |
| 12 | TxEnhW | Transcribed and Weak Enhancer | Electric Lime | 194,225,5 |
| 13 | EnhA1 | Active Enhancer 1 | Orange | 255,195,77 |
| 14 | EnhA2 | Active Enhancer 2 | Orange | 255,195,77 |
| 15 | EnhAF | Active Enhancer Flank | Orange | 255,195,77 |
| 16 | EnhW1 | Weak Enhancer 1 | Yellow | 255,255,0 |
| 17 | EnhW2 | Weak Enhancer 2 | Yellow | 255,255,0 |
| 18 | EnhAc | Primary H3K27ac possible Enhancer | Yellow | 255,255,0 |
| 19 | DNase | Primary DNase | Lemon | 255,255,102 |
| 20 | ZNF/Rpts | ZNF genes & repeats | Aquamarine | 102,205,170 |
| 21 | Het | Heterochromatin | Light Purple | 138,145,208 |
| 22 | PromP | Poised Promoter | Pink | 230,184,183 |
| 23 | PromBiv | Bivalent Promoter | Dark Purple | 112,48,160 |
| 24 | ReprPC | Repressed Polycomb | Gray | 128,128,128 |
| 25 | Quies | Quiescent/Low | White | 255,255,255 |

We define enhancers as the union of states #10-18. This definition is intended to include as many active enhancers as possible, while excluding other types of regulatory elements that are defined by ChromHMM states. For instance, we specifically exclude transcription start sites, promoters, repressed regulatory regions (e.g., State 24, “Repressed Polycomb”), and harder-to-define regions with regulatory potential such as State 19 “Primary DNase”. The model does not have distinct categories for silencers and insulators, so it is not entirely clear how these elements would be distributed across states. We have clarified these points in the Methods section on “Enhancer download and processing”. Additionally, we have performed a new analysis with the brain chromHMM enhancers looking for corroboration of the enhancer calls using an independent dataset. We overlapped ChIP-seq for the enhancer marker H3K27ac and the heterochromatin marker H3K9me3 from human brains (middle frontal area 46) and found that the brain enhancers are significantly enriched for H3K27ac and depleted for H3K9me3 as we would expect. This analysis is available as Supplemental figure 6 and is detailed in Methods section 5.

R1) 3. On page 14, the authors used MPRA and eQTL data to support a few top RWAS signals. I think it is more important to have an overall evaluation, like an overlap or enrichment test, to show how many or % of RWAS signals can be validated. eQTL is particularly interesting.

Thank you for this suggestion. To address this issue, we conducted a new RWAS analysis to test this more rigorously. We found that enhancers associated with risk for SCZ are more likely to overlap brain eQTLs than enhancers that do not overlap brain eQTLs. This result suggests that SCZ-associated enhancers are among those that most directly impact gene expression in the brain. We have added this new analysis in Results section 2, paragraph 4.

Reviewer 2

R2) 1. I find the title a little confusing. It would be easier to read if it specified that RWAS is a method for identifying those properties. Additionally, the paper seems much more focused on downstream findings than the RWAS method itself.

We have changed the title to “Identification of enhancer properties associated with genetic risk for complex traits with regulome-wide association studies” in an effort to make the focus more clear to the reader.

R2) 2. Figures S1 E&F should say average enhancer length.

This has been changed in the figure.

R2) 3. Is there a good reason why fetal female and fetal male don’t cluster together (Figure 2)? Would be good to discuss this in the text.

Thank you for bringing this up. Indeed, while the female fetal brain and male fetal brain were in the same cluster, the female fetal brain enhancers were slightly more similar to the neurosphere samples than to the male fetal brain samples. This is likely due to technical differences, as fetal male brain (E081) is the only sample from this cluster where the ChIP-seq was performed at the Broad Institute, while all other neurosphere/fetal samples were from UCSF. The differences in sample origin did not have a major effect on the overall cluster structure, as E081 did not cluster with any of the other Broad Institute samples such as H9 derived neuronal progenitor cultured cells (E009), H9 derived neuron cultured cells (E010), or NH-A Astrocytes (E125). We have included this discussion in the legend of Supplementary figure 3.

R2) 4. Looking at Figure 3b, there appear to be brains with nearly identical levels of significance which is surprising. Is there a good reason for this?

Upon close examination, the very similar appearing p-values in Figure 3B are not identical (e.g., P = 3.50E-09 vs 4.90E-09). We do not believe there is any underlying issue with the calculation of the p-values. To improve clarity, we have added a Supplementary Table 4 with the exact p-values.

R2) 5. Figures 4a and b are informative, but they are not well described in the text.

We have updated the figure description in the text.

R2) 6. It is claimed that “Examination of specific risk loci indicated that risk-associated enhancers capture the genetic risk signal at many of the SNP-based risk loci in a tissue-specific manner” [line 197] but In this example I also see significant schizophrenia risk in enhancers in primary t-cells and not temporal lobe. Is this enhancer also a true positive? Furthermore, it would be good to have some summary statistics of hits in enhancers across tissues (e.g. fraction of genome-wide significant hits captured by each enhancer set).

Thank you for bringing this observation to our attention. On the whole, brain-specific enhancers were more strongly associated with SCZ risk than enhancers in other tissues. However, as the reviewer points out there are indeed a small number of SCZ risk loci at which we found more significant enhancers captured by the T-cell enhancer annotations than by the brain enhancer annotations. The loci that were better reflected by the immune annotation could certainly be reflective of pathophysiological mechanisms mediated by immune cells, though we cannot rule out false positives. We have added a list of genome-wide significant enhancer hits within SCZ risk loci across all 127 tissues to facilitate comparison (Table S3).

R2) 7. The MPRA analysis is quite ad-hoc. It needs numbers for how many overlapping hits were observed and how many would be expected.

We thank you for the suggestion, and we have added a new analysis comparing the number of significant MPRA hits in our fetal male brain enhancers versus shuffled genomic regions with similar characteristics, focusing on enhancer annotations from the male fetal brain. We found that fetal brain-expressed enhancers contained nearly twice as many significant MPRA SNPs as would be expected in a set of shuffled genomic regions with the same characteristics (permutation p-value < 0.05 across 10 brain enhancer sets). These results are detailed in Results section 2 paragraph 3 and the methods are described in Methods section 5.

R2) 8. In Figure 5a, do stars represent significance? Also, how was this subset of the 64 gene sets selected? It would be good for some gene sets that would be expected to be non-significant to be shown for comparison as negative controls.

Yes, stars indicate significance. This has been clarified in the figure legend.

R2) 9. Figures 6a and 6b, should show some other GO terms for comparison. A negative control would also be good.

We agree that GO term enrichments are difficult to difficult without a null hypothesis term for comparison. A new supplementary figure (Supplementary figure 7) comparing the result to general ontology terms “biological function,” “cellular compartment,” and “molecular function” has been added to facilitate comparison to non-significant enrichments.

R2) 10. In Figure 6d, is this the full set of TFs that recognize positively associated motifs? If not, how were they selected? Also, it's hard to tell by looking at the figure if expression levels are significant.

The TFs shown in this figure recognize a motif with an RWAS Z-score > 3 in a brain enhancer, and we have clarified these selection criteria in the figure legend. We did not perform a significance test in this analysis, so the figure should be interpreted as a description of the diversity of cell type-specific expression patterns for these TFs.

Review 3

R3) 1. There seems to be no new code/method developed here (h<ttps://github.com/casalex/RWAS)>, simply commands to run MAGMA for this application. Please rewrite the paper to make it clear this is not a novel computational method but just a new application of MAGMA.

We have added language clarifying that this framework is an implementation of MAGMA.

R3) 2. The proposed approach is based on running MAGMA. However, MAGMA is developed for studying genes. Utilizing same approach for application to enhancers might have some unforeseeable complications. For example, the fact that multiple enhancers can have complicated non-linear relationships with gene expression of multiple genes. How would this impact the result of the method?

The non-linear relationships between enhancer activity and gene expression are a fascinating yet complicated aspect of gene regulations, and we agree that this is challenge for methods that attempt to aggregate all of each gene’s enhancers into a combined score (e.g., H-MAGMA). To avoid this issue, we designed RWAS to utilize p-values for individual enhancers rather than attempting to aggregate the effects of all enhancers that may interact with a particular gene. Many of the enhancer properties that we consider in our analysis (e.g., evolutionary conservation, GC%) do not rely on an annotation to a gene at all. In analyses that do rely on enhancer-gene mapping (e.g., GO term enrichment) we consider all of the genes that are predicted to interact with each enhancer, accounting for the complexity of these relationships. In summary, we considered these issues carefully in the design of our method. We have re-written portions of the introduction (lines 88-131) to make sure these points are clear.

R3) 3. There are several methods developed that also calculated the association of enhancer with diseases (e.g., FENRIR or ABC model). Please do a comparison with other computational methods.

We conducted new analyses comparing RWAS to FENRIR. We find that RWAS and FENRIR identify many of the same regions as top-scoring enhancers associated with SCZ. RWAS has several unique features that distinguish it from FENRIR. Notably, the ChromHMM models provide a more extensive catalog of regulatory regions in the human genome (FENRIR networks were based on just ~48,000 enhancers, whereas ChromHMM models describe hundreds of thousands across human tissues). Also, leveraging MAGMA gene property analysis makes it straightforward to use RWAS to calculate associations with enhancer features, which may be more difficult to discern from FENRIR models. We have added a new section (Results section 2 paragraph 2) describing the comparison between FENRIR-predicted enhancers vs. RWAS, and we added a description of FENRIR in the paragraph on existing methods (lines 104-105).

The ABC model is primarily a method to predict the target genes of enhancers. As mentioned above, this strategy is quite distinct from RWAS, which tests for associations with the enhancers themselves and is not concerned with enhancer—target gene interactions. We have added a description of ABC to the Introduction, as part of our discussion of existing methods (lines 96-97). A head-to-head comparison is not feasible, since ABC and RWAS address different questions.

R3) 4. There are well known CNVs correlated with schizophrenia. Is there an enrichment of these CNVs impacting the predicted enhancer?

We tested for overlap of schizophrenia-associated CNVs with enhancers using a set of 12 CNV regions collated by Warland et al. 2020 (PMID: 30679740). Not surprisingly, given that CNV regions are large, we found that there are many enhancers within the bounds of schizophrenia-associated CNVs. However, within the context of a large CNV this does not on its own provide much benefit for fine-mapping the causal enhancers and genes. We therefore considered the possibility that enhancers overlapping schizophrenia-associated CNVs represent a class of enhancer that is enriched for low p-values from SCZ GWAS. We performed a RWAS analysis treating overlap with a known schizophrenia-associated CNV as a binary independent variable, testing for an enrichment of risk in enhancers located in these CNVs. We did not find any significant associations in this analysis, most likely because there are only 12 schizophrenia-associated CNVs and their overlap with GWAS risk loci is modest. We have chosen not to include this analysis in the manuscript, as our primary focus is on the interpretation of GWAS. However, we added a sentence to the Discussion mentioning fine-mapping of CNV regions as a potential future direction. Discussion, 2^nd^ to last paragraph: “While our current approach is focused on testing associations of enhancers with common SNPs identified through GWAS, annotating gene regulatory consequences of rare non-coding single-nucleotide variants and copy-number variants represents an important future direction.”

R3) 5. There are studies that have shown significant enrichment of denovo coding variants in SCZ cases (Gulsuner et al. 2013). Can the author find some sequencing data that shows significant enrichment of rare variants in affected cases impacting these enhancers?

Annotating rare non-coding variants, including de novo variants, is an important goal for the field. Unfortunately, it has not been possible to address this yet for schizophrenia, since most of the published studies of rare variants in schizophrenia used exome sequencing, which does not provide coverage of enhancer regions. As large-scale whole-genome sequencing datasets for schizophrenia become available, we look forward to addressing this as in the future. We mentioned above, we have added a sentence to the Discussion mentioning this as a future direction: “While our current approach is focused on testing associations of enhancers with common SNPs identified through GWAS, annotating gene regulatory consequences of rare non-coding single-nucleotide variants and copy-number variants represents an important future direction.”
