# Supplementary material for: Identifying enhancer properties associated with genetic risk for complex traits using regulome-wide association studies
Source: PLoS Comput Biol. 2022 Sep 7;18(9):e1010430. doi: 10.1371/journal.pcbi.1010430 (PMC9484640; doi:10.1371/journal.pcbi.1010430)
Supplement: Attachment — Submitted filename: reviewer_comments_response_v4.docx [file pcbi.1010430.s013.docx]

**Responses to reviewers**

Thank you for the opportunity to respond to the critiques of our manuscript “Identifying enhancer properties associated with genetic risk for complex traits using regulome-wide association studies.” We thank the reviewers for their valuable feedback and have addressed each of their concerns, as described below.

Reviewer 2

R2) The authors raise an interesting point about the fetal female brain sample. Given the technical differences, I wonder if it may be worth excluding that sample. I leave it up to the authors.

The reviewer’s point is well taken, and we agree that technical differences may make direct comparison of the male and female fetal samples more complicated. However, we feel that this sample is a valuable one to have in our dataset, and that the technical differences are somewhat unavoidable in samples derived from large consortia such as this one. We thank the reviewer for their suggestion that we provide a discussion in the text of these differences, as we feel that its addition has made it a more useful resource and a more thoughtful manuscript.

R2) line 222 - “male fetal brain” is missing the word “enhancers”

Thank you, we have corrected this in the text.

R2) Regarding figure 5a, I still don't see how a subset of the 64 were selected.

We apologize for the omission in our previous revision, and a description of the selection criteria has been added to the figure legend.
